# The influence of experience on contest assessment strategies

**DOI:** 10.1038/s41598-017-15144-8

**Published:** 2017-11-03

**Authors:** Irene Camerlink, Simon P. Turner, Marianne Farish, Gareth Arnott

**Affiliations:** 1 Animal Behaviour & Welfare, Animal and Veterinary Sciences Research Group, Scotland’s Rural College (SRUC), West Mains Rd, Edinburgh, EH9 3JG UK; 2 Institute for Global Food Security, School of Biological Sciences, Queen’s University, Belfast, BT9 7BL UK

## Abstract

Animal contest behaviour has been widely studied, yet major knowledge gaps remain concerning the information-gathering and decision-making processes used during encounters. The mutual assessment strategy, where the individual assesses its own fighting ability (Resource Holding Potential, RHP) and compares it to that of its opponent, is least understood. We hypothesise that individuals need experience of agonistic encounters to become proficient at mutual assessment. Pigs (*Sus scrofa*, n = 316) were contested twice. In between contests, animals did or did not (control) receive intense fighting experience. A substantial proportion of the contests reached an outcome with a clear winner without fighting. Non-escalation was highest in RHP asymmetric dyads of the second contest, irrespective of experience. In contest 1 (no experience) and in contest 2 for the experienced animals, costs increased with loser RHP and where unaffected by winner RHP, suggesting a self-assessment strategy. In contest 2 control dyads, which only had experience of one prior contest, a negative relation between winner RHP and costs suggested mutual assessment during the pre-escalation phase but not during escalated aggression. This reveals that a brief and relatively mild experience can be beneficial in the development of mutual assessment whereas profound experience may result in adoption of a self-assessment strategy.

## Introduction

Throughout the animal kingdom access to limited resources may lead to contests, mediated by various forms of agonistic behaviour. The unequal distribution of resources arising from agonistic encounters directly impacts fitness, driving natural selection^1^ and sexual selection^2^. Despite the importance of animal contest behaviour, major knowledge gaps remain concerning the information-gathering and decision-making processes used during encounters^3–5^. Important asymmetries exist between contestants including fighting ability, termed resource holding potential (RHP; ref.^6^), resource ownership, and the value of the resource to each contestant^7^. Selection is expected to favour contestants that gather information about such asymmetries and use that to inform decision making^3,7^. Game theory models have provided a useful framework to further our understanding of animal contest behaviour, and since the original hawk-dove game^8^, that involved no information-gathering, a suite of more realistic models have been developed that differ in the assessment strategies used. A related major knowledge gap concerns how animals develop and acquire the social skills to make appropriate assessments during contests. Specifically, we hypothesise that animals may require experience of multiple agonistic encounters to become proficient at mutual assessment (assessment of relative RHP difference between opponents).

Contest theory models can be grouped into three main types that differ in the information about RHP that opponents are presumed to gather. The first, termed pure self-assessment, is a feature of the ‘war of attrition without assessment’ (WOA-WA; ref.^9^) and energetic war of attrition (E-WOA; refs^10,11^). Here, each contestant has information about its own RHP but gathers no information about the opponent. Rivals persist in line with their own RHP, with the accumulated costs only relating to their own actions. Inferior opponents will reach their limits first and give up. The second assessment strategy is encompassed by the cumulative assessment model (CAM; ref.^12^), and is also a form of self-assessment. However, in contrast to pure self-assessment, in the CAM costs also accumulate from the opponent’s actions. This means that in the CAM the decision to withdraw is influenced by both an individual’s own RHP, with weak rivals capable of bearing fewer costs, and also the opponent’s RHP, with higher quality individuals inflicting costs at a higher rate. The third model is mutual assessment, which involves an assessment of relative RHP difference between opponents. This is generally interpreted as an individual gathering information about the fighting ability of a rival and comparing this against an assessment of their own ability. This form of assessment is central to the ‘sequential assessment model’ (SAM; refs^13,14^) and the ‘asymmetric war of attrition’ (AWOA; refs^15,16^), with the selective advantage that the weaker rival can terminate the contest, minimising costs, as soon as it perceives its inferiority. However, the majority of previous studies supporting mutual assessment were shown to have used inappropriate analyses that could not distinguish it from pure self-assessment and CAM^17^. Since the publication of a review^3^ summarising these issues and providing researchers with the correct approaches to use, there has been a resurgence of interest in this area. To discriminate between the alternative assessment strategies it is necessary to examine relationships between individual contestants RHP and contest cost^3^. All models predict a positive relationship between loser RHP and contest cost (typically measured as contest duration). Therefore it is important to examine the relationship between winner RHP and contest costs, with pure self-assessment predicting a weak positive or non-significant relationship, while mutual assessment and CAM predict this relationship to be negative^3^. To discriminate between mutual assessment and CAM it is necessary to examine contests in which opponents are matched for RHP, with CAM predicting a positive relationship between the average RHP of matched pairs and contest cost, while no such relationship is predicted for mutual assessment^3^. To date, few studies provide clear evidence for mutual assessment (although see ref.^18^ for a comprehensive example of mutual assessment in cuttlefish). Despite this, mutual assessment retains intuitive appeal, perhaps because of our human aptitudes for this strategy^19,20^.

We hypothesize that individuals may require experience of agonistic encounters to be able to assess an opponent’s fighting ability, as in mutual assessment. To date, while studies have investigated the role of experience on fight outcome, identifying so-called winner and loser effects^21^, to our knowledge no studies have investigated how experience influences contest assessment.

This hypothesis was addressed in pigs (*Sus scrofa domesticus*). Domestic pigs allow for a controlled experimental set-up in which genetics and life history are known, whereas, when released to nature, their behaviour soon reverts to the natural behaviour as shown by their ancestors the wild boar^22^. Pigs have a broad spectrum of agonistic behaviour, ranging from very subtle ritualized display to long escalated fights, and have been assumed to be capable of mutual assessment^23,24^. Because of the welfare implications of pig aggression under commercial husbandry conditions, their aggression has been well studied, including through the use of contest theory models^25,26^.

Contests between pigs include various phases of escalation^26^. Animals may switch assessment strategy between different contest phases. For example, killifish (*Kryptolebias marmoratus*; ref.^27^) use mutual assessment during initial phases, switching to self-assessment during an escalated phase^27^. The extent to which this occurs in other species remains to be investigated. Ignoring potential differences between phases may result in false conclusions about the assessment strategy in use and may overlook, or falsely assume, the occurrence of mutual assessment. This study will therefore also examine whether contestants switch assessment strategies between phases and whether this interacts with experience, with the hypothesis that more experienced individuals will sooner switch to mutual assessment.

In addition, various contest costs are measured. In species that by nature aim to avoid damaging behaviour, and instead use ritualized display, the total contest duration may not reflect the actual costs when compared to contests that do escalate into damaging aggression but are shorter in duration^26^.

This study aims to determine what RHP assessment strategy is used during contests between pigs that have never previously met an unfamiliar conspecific, and how experience of fighting affects these strategies in later contests. This will investigate the prediction that pigs possess the capacity for mutual assessment but that experience of fighting is necessary to become proficient at this.

## Methods

### Ethical note and justification of sample size

This study was approved by SRUC’s animal experiments committee and was carried out under UK Home Office license (project licence PPL60/4330), and in constant collaboration with SRUC’s named veterinary surgeon. The study was carried out in accordance with the recommendation in the European Guidelines for accommodation and care of animals, UK Government DEFRA animal welfare codes, and adhered to the ASAB/ABS guidelines. Strict end-points were in place for the termination of contests, ensuring that the welfare of the animals was not compromised. This prevented any injury other than skin lesions due to receiving bites.

The sample size was determined based on the treatment design (described in ‘*Experimental design*’) equating to a 2 × 2 × 2 design. The minimum amount of dyads per treatment group was set to 15 (n = 30 pigs) which needed to be balanced for sex and aggressiveness as a personality trait and needed to guarantee that none of the animals encountered a same conspecific twice on the three staged encounters (other than their siblings). Based on previous work^25^ we accounted for a 40% chance of non-escalation and 10% chance of contests without a clear outcome that could limit the use of the data of those contests. This resulted in an aimed sample size of 360 pigs, which resulted in a slightly lower sample size of 316 due to a lower number of piglets born from the allocated sows.

### Animals and housing

A total of 316 male and female pigs (a commercial type cross of a Large White × Landrace sow serviced by an American Hampshire boar) were studied until 13 weeks of age at the SRUC pig research farm (Easter Howgate, UK). The animal phase was conducted over four consecutive batches from Nov 2014–Nov 2015. Piglets had been raised in conventional farrowing crates. Males were not castrated and the tail and teeth were kept intact. Piglets remained in their own litter. Piglets were weaned from the sow when they were four weeks of age. After weaning they were kept in the same litter group but moved to a pen measuring 1.9 × 5.8 m, allowing ~1.0–1.1 m^2^ per pig. Pens had a solid floor which was covered with approximately 5 kg of long straw. Pens were cleaned daily and provided with ~3.5 kg of fresh straw. Pigs had *ad libitum* access to water and pelleted commercial feed. From 6 to 8 weeks of age pigs were habituated to the various test situations (described below) to reduce the likelihood of a fear response during the tests and procedures. Habituation involved gradually exposing pigs to being alone in a known and unknown area for several minutes and to being handled in a weigh crate. At 9 weeks of age each pig was tested twice in a resident-intruder (RI) test to gain an individual estimate of aggressiveness as a personality trait^28^. This test (described in detail in^25^) measures the latency to attack an inferior intruder. The correlation between the attack latencies of both test days was weak but significant (*r* = 0.26; *P* < 0.001), in contrast to previous work (ref.^29^: *r* = 0.55–0.73), including on the same population of pigs (ref.^26^: *r* = 0.58; *P* < 0.001). The two test values were summed to obtain a single measure of aggressiveness (as in ref.^28^).

### Experimental design

**Figure 1:**
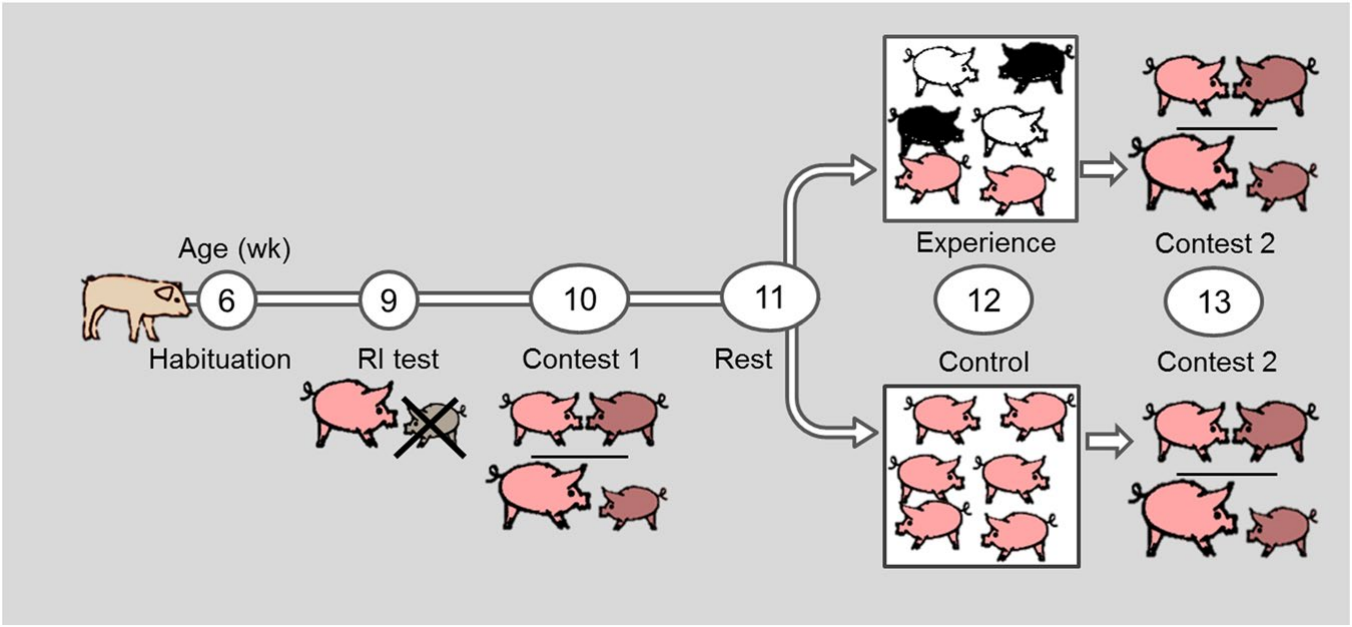
Experimental design. Graphical presentation of the various tests by week of age.

Details of each of the procedures are given below. Briefly, pigs, naïve to encountering unfamiliar conspecifics (besides the very brief RI test of <5 min in which they encountered but did not fight an inferior pig), were first tested at 10 wk age in a dyadic contest to determine their assessment strategy without experience. Two weeks later, at 12 wk age, 55% of the study population was subjected to group mixing which involved repeated fights. This simultaneous encounter with several unfamiliar conspecifics rapidly increases pigs’ experience in fighting. At 13 wk of age, each pig was tested a second time in a dyadic contest to formally test assessment strategy. A timeline of the experimental design is given in Fig. 1. Contest costs were measured by contest duration, fight duration, and changes in the number of skin lesions, blood lactate and blood glucose (details described below). Note that ‘contest’ and contest duration refer to the full time from opponents entering the arena until exiting the arena, whereas ‘fight’ and fight duration refer only to the time when opponents are mutually attacking each other with bites within the contest.

### Contest 1

Contests were staged between pairs of pigs at 10 wk of age. Pigs were randomly matched with an opponent of either similar body weight (RHP matched; <5% weight difference) or varying body weight (RHP asymmetry; >20% weight difference) in order to maximise variation in relative weight. Before pairs were randomly matched, the distribution of males and females and variation in attack latency as measured in the RI test was balanced between treatments. Contests were staged in a novel and neutral test arena measuring 2.9 × 3.8 m. The arena had a solid floor with a light bedding of wood shavings. There were no resources present in the arena. The opponents entered the contest arena simultaneously from opposite sides. The time was started from the moment both had entered the arena fully. Contests were ended when a) a clear winner was apparent; b) after 30 minutes without a clear winner; or c) in the event that a fight or mounting behaviour became too severe or that the animal showed repeated fear behaviour. The determination of a winner was based on the retreat of one pig (the loser) without retaliation for 1 min. In total 157 contests were carried out. Ten contests (6%) reached an end-point due to a fear response or mounting, and in five contests (3%) the maximum time was reached without an established winner.

### Experience of aggression

At 12 weeks of age (two weeks after contest 1), 55% of the tested pigs were mixed into a new group with unfamiliar pigs to gain experience of aggressive interactions (the percentage being based on equal sized groups in the group mixing). The remaining 45% served as a control group and were by pen (only siblings together) relocated into smaller pens to maintain a similar space allowance per animal while the group size was reduced (due to the removal of pigs for the experience treatment). Control pigs did not encounter any unfamiliar pigs. The mixed groups consisted of three pairs of pigs of mixed weights, originating from different litters so that each pig was familiar to one pig but unfamiliar to four. The inclusion of a familiar pig in the new group was designed to prevent pigs from becoming too distressed, whereas the four unfamiliar pigs were expected to induce an aggressive reaction. Pigs were left undisturbed for the first 24 h after mixing, after which aggression commonly subsides. Pigs remained within this group composition for the rest of the trial to avoid further disruption of dominance relationships. Skin lesions were counted as a reflection of the intensity of engagement in aggression (following^30^). Counting took place in the morning before mixing and 24 h after mixing, both on the regrouped animals and on the control animals.

### Contest 2

At 13 weeks of age all pigs were matched for a second contest to determine how fighting experience influenced assessment ability. Contest 2 was executed as described for contest 1, but with a 2 × 2 treatment design including body weight (matched/asymmetric) and experience of group mixing (control/experienced). Pigs were paired with an opponent they were unfamiliar to, which meant that opponents were not from the same litter and had not encountered each other previously in either contest 1 or during group mixing. Contests were only staged between pigs with similar experience level (control/experienced). Similar to the first contest, blood metabolites and skin lesions were recorded. For contest 2 not all pigs could be matched with an unfamiliar opponent of the same treatment group, and therefore the number of contests reduced to 154. Of these, 30 (10%) reached an end-point due to fear or mounting, and nine (6%) reached the maximum time with no winner (30 min).

### Blood glucose, blood lactate and skin lesions

Blood glucose and blood lactate values were obtained within 3 min pre- and post-contest. A drop of blood was collected from the ear vein by a pin prick which was taken when the pig was located in a weigh crate. The drop of blood was then immediately applied to a test strip on a handheld glucose meter (IME-DC iDia) and lactate meter (The EDGE Lactate Analyser) developed for humans. This method was previously applied with success^25^. Sampling order was randomized for treatment group and contest outcome. The proportional increase in blood value (post-test value/pre-test value) was used for analyses. Skin lesions, which are scratches on the body as a result of receiving bites, were counted in the morning before testing and directly after the contest by a single observer.

### Behavioural observations

The latency until the first contact, first bite, first fight and final retreat was recorded live during the contests by one observer who was blind to the treatments. The latency until the first fight, or the latency until final retreat in the case of no fight, was used to distinguish a pre-escalation phase. Contests were recorded on video and were analysed for the exact fight duration using The Observer XT 10 (Noldus, The Netherlands). Fighting was defined as an aggressive act, e.g. biting and pushing, which the recipient retaliated to with an aggressive act within 5 s, and continued until one opponent retreated or until other behaviour was performed for at least 3 s. The duration of the pre-escalation phase and the fight duration (escalation phase) were used to investigate whether pigs switched between assessment strategies during the contest, by analysing the assessment strategy over the duration of the pre-escalation phase and escalation phase separately.

### Statistical analyses

First, descriptive statistics for all of the contests were investigated. Then, contests without a clear winner (time-out or end-point) were excluded. Contests where an endpoint occurred within the minute after an outcome was reached were included (e.g. when repeated mounting occurred within one minute after final retreat). Data were analysed with SAS version 9.3 (SAS Institute Inc., Cary, USA). Results are presented as LSmeans with standard error unless stated otherwise.

#### Model assumptions

Residuals of the continuous response variables were assessed for the normality of the distribution (UNIVARIATE Procedure, Shapiro Wilk statistics) and outliers (using Studentized residuals). Contest duration, the pre-escalation duration and the fight duration were skewed and were log transformed to reach a normal distribution. The number of skin lesions were square root transformed (sqrt) to reach normality of the residuals. All models were tested for multicollinearity (REG Procedure; VIF option), independence (REG Procedure; Durbine Watson option), and homoscedasticity (AUTOREG Procedure; Arch option). The variance components covariance structure (VC; default in SAS) best fitted the models as assessed through the Akaike Information Criterion (AIC) and the Bayesian Information Criterion (BIC). All models were specified based on the best model fit as assessed through the AIC and BIC.

#### Analyses on the individual level

Basic statistics were calculated on the individual level as most of the measurements were obtained per individual. To select the most suitable measures of contest cost for further analyses, Pearson correlations were estimated between contest duration, fight duration, the number of skin lesions, blood lactate and blood glucose. Based on those correlations (section ‘*Results – Measures of contest costs*’) the variables contest duration (as traditional measure), fight duration, and skin lesions were retained. Analyses of fight duration excluded those contests in which no fight occurred. Differences in the occurrence of escalation between contests and treatment groups were analysed through contingency tables with Chi Square analysis. Paired t-tests were applied to test the differences between winners and losers in terms of the number of skin lesions and the body weight (contest 1 and 2 analysed jointly).

#### Analyses on the dyad level

Only three variables that were measured on the individual level were retained in the further analyses (these were body weight, skin lesions and attack latency). Further analyses were therefore carried out at the dyad level, with the three pig-level variables separated by winner and loser within a dyad (e.g. winner lesions, loser lesions). Assessment strategy is traditionally analysed by the direction of the relationship between fighting ability (RHP) and the contest costs (e.g. contest duration) for winners and losers separately^3^. RHP matched and RHP asymmetric dyads were analysed jointly (as a linear scale of RHP difference). RHP difference was initially included in all models as a fixed effect but was omitted as it did not significantly affect the contest costs. RHP difference did affect escalation level and therefore these statistics are presented by RHP matched versus asymmetric dyads. General Linear Mixed Models (MIXED Procedure) were run for the response variables contest duration, the duration of the pre-escalation phase, fight duration (escalation phase), winner skin lesions, and loser skin lesions. The strength and direction of the slope of contest costs against winner and loser RHP were assessed through the three-way interaction between contest number (1/2), experience (contest 1/contest 2 control/contest 2 experienced) and RHP, for both winner and loser separately in the same model. This three-way interaction at the same time allowed to assess the differences between the slopes of the treatment groups. The combination of sexes in the dyad (MM/FF/MF) was included as fixed effect. Winner and loser aggressiveness was initially included as a covariate in all models as it has previously been shown to affect contest behaviour (^25,26^), but was excluded as it was non-significant and reduced the model fit. Batch (group tested in the same week) was included as the random effect. Beta values are back-transformed LSmeans.

### Data availability

The datasets generated during and/or analysed during the current study are available on request from the corresponding author.

## Results

### Measures of contest costs

The correlations between the various measures of contest costs reveal that fight duration and skin lesions, both in contest 1 and in contest 2, best captured the total contest costs in terms of duration, fatigue and injury (Table 1). In contest 1 fight duration correlated at greater than *r* = 0.50 with all other measures of contest cost, and in contest 2 between *r* = 0.38 and *r* = 0.80. The number of skin lesions, which reflects each bite that an animal had received, was an equally good measure. However, skin lesions can be measured in each contest on a continuous scale whereas fight duration can only be applied in contests in which an escalated fight occurred. Skin lesions also provide a distinction in costs between the winner and the loser, with losers having on average double the number of skin lesions as compared to winners (winners: 32 ± 3 lesions; losers: 61 ± 4 lesions; *t*_279_ = −9.89, *P* < 0.001). Based on the correlations in Table 1 we continued the analysis with only the duration of the pre-escalation phase, the fight duration and the number of skin lesions for winners and losers.

**Table 1:** Proxy measures of contests costs.

	Contest duration	Fight duration	Blood lactate	Blood glucose	Skin lesions
Contest duration		0.51	0.20	0.25	0.36
Fight duration	0.61		0.62	0.50	0.70
Lactate	0.37	0.61		0.58	0.49
Glucose	0.19	0.38	0.51		0.45
Skin lesions	0.55	0.80	0.59	0.37	

Pearson correlation coefficients between various proxy measures of contest costs for contest 1 (values above the diagonal) and contest 2 (values below the diagonal). All correlations are significant at *P* < 0.001.

### Contest escalation

Depending on the treatment group, between 37 to 74% of the contests with a clear winner did not escalate into fighting (Table 2). Instead, in these contests dominance relationships were established through milder forms of agonistic behaviour, such as a single bite followed by immediate retreat.

**Table 2:** Non-escalation.

	Contest 1	Contest 2 Control	Contest 2 Experienced
RHP Matched	37^a^ (25/68)	56^b^ (19/34)	45^b^ (19/42)
RHP Asymmetric	39^a^ (29/75)	73^b^ (22/30)	74^b^ (23/31)

Values are the percentage of contests that reached an outcome (clear winner) without fighting. The number of contests out of which the percentage is calculated is presented in parentheses. ^a,b^Values lacking a common superscript letter differ by *P* < 0.10.

The highest number of contests without escalation, 74%, occurred in RHP asymmetric dyads of contest 2, irrespective of whether they had undergone the regrouping experience or not (Table 2; asymmetric dyads: contest 1 vs. contest 2 control: χ^2^(1) = 10.3, *P* = 0.002). Overall, the percentage of non-escalation was higher in contest 2 as compared to contest 1 (χ^2^(1) = 14.58, *P* < 0.001). Within contest 2, the inexperienced (control) group did not differ from the experienced group (χ^2^(1) = 0.61, *P* = 0.49). RHP asymmetric dyads tended to escalate less than dyads in which the opponents were matched (χ^2^(1) = 3.18, *P* = 0.09). Matched dyads tended to escalate more in C2 than in C1 (Table 2; C1 vs. C2 control: χ^2^(1) = 4.03, *P* = 0.06). Due to the absence of a fight in some of the contests, the fight duration (i.e. escalation phase) was analysed only for 89 dyads in contest 1 and for 54 dyads in contest 2 (control n = 23; experienced n = 31).

### Assessment abilities in contest 1

**Figure 2:**
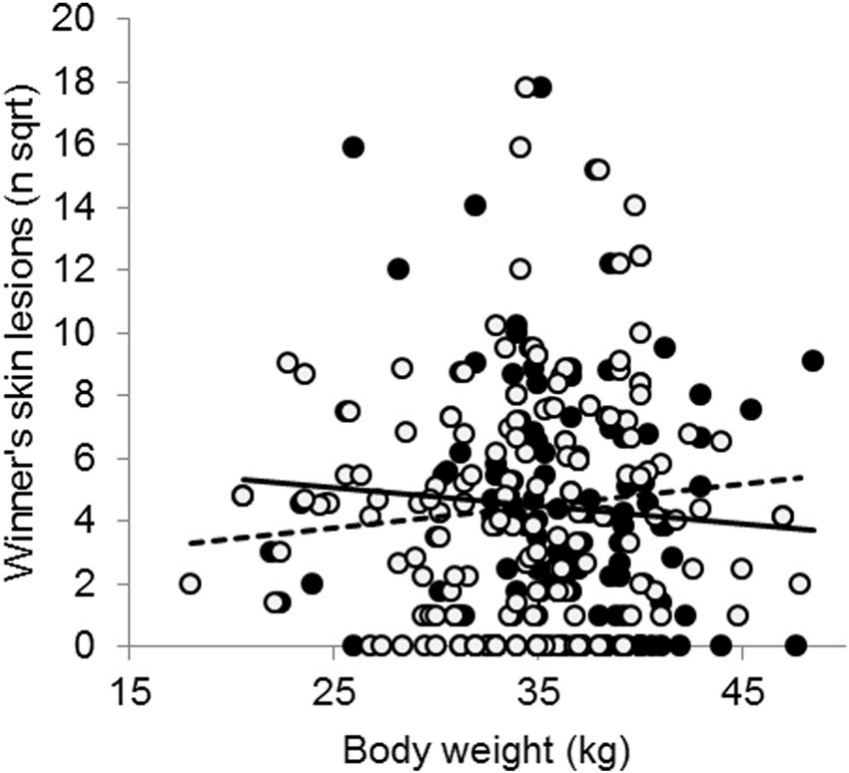
Assessment strategy before experience. The relationship between winner and loser body weight for skin lesions on the winner’s body as measure of contest costs in contest 1. Winners (n = 135): ●/−; Losers (n = 135): ○/---.

Body weight was used as a proxy measure of fighting ability (RHP). Indeed, across contests the heavier opponent was more likely to win (winner: 46 ± 0.7 kg; loser: 44 ± 0.7 kg; *t*_277_ = 4.71, *P* < 0.001). In contest 1, when none of the pigs had encountered an unfamiliar pig before, the contest duration increased with loser RHP (*b* = 10 s/kg; *t*_267_ = 1.97; *P* = 0.05). Likewise, the number of skin lesions on the winner’s body increased with the increase of loser RHP (Fig. 2; *b* = 21.6 lesions/kg; *t*_268_ = 2.85; *P* = 0.005). Pre-escalation duration, fight duration and the number of lesions on the loser were unaffected by loser RHP and none of the measures were significantly affected by winner RHP. Thus, stronger losers inflicted more injuries on the winner than weak losers, irrespective of the size of the winner.

### Experience of regrouping aggression

Skin lesions on the body, which are a reflection of the number of bites received, provide information on the amount of engagement in fights. Pigs undergoing the regrouping experience had on average 124 ± 89 skin lesions on their body. In contrast, control pigs (i.e. those that had not been regrouped but were relocated and had a change in group composition due to the removal of group mates) had only 7 ± 10 skin lesions. Although this indicates that control pigs did bite their siblings, the intensity as reflected by the mean number of skin lesions was negligible in comparison with the regrouped pens (lesions control vs. experienced: *t*_135_ = 23.31; *P* < 0.001).

### Assessment ability after experience

In control dyads, which had experience of contest 1 but no profound fighting experience, the duration of the pre-escalation phase was influenced by winner and loser RHP indicative of mutual assessment. The pre-escalation phase increased with loser RHP (*b* = 10 s/kg; *t*_265_ = 2.38; *P* = 0.02) and decreased with increasing winner RHP (*b* = −10 s/kg; *t*_265_ = −2.21; *P* = 0.03). All other measures of contest costs were not significantly affected by winner and loser RHP.

Animals that had received profound fighting experience had a longer contest duration than control dyads, which was due to a longer pre-escalation phase (Table 3). As this was due to more non-damaging behaviour, as the number of injuries did not differ between the control and experienced dyads (Table 3). In experienced dyads, the number of skin lesions on the winner’s body increased when loser RHP increased (*b* = 14.2 lesions/kg; *t*_268_ = 2.19; *P* = 0.03) but was unaffected by winner RHP. The number of skin lesions on the loser’s body, in line with winner lesions, also increased with loser RHP (*b* = 21.8 lesions/kg; *t*_268_ = 2.07; *P* = 0.04) but was unaffected by winner RHP. In other words, the stronger the loser was the more injuries it delivered to the winner, irrespective of the winner’s size, but in addition this also resulted in the loser receiving more injuries in return. Winner and loser RHP did not affect the contest duration, pre-escalation duration, or fight duration.

**Table 3:** Contest costs.

	Contest 1	Contest 2 Control	Contest 2 Experienced	*P*-value
Contest duration (s)	263 (184–342)^a^	159 (107–211)^b^	209 (140–278)^a^	0.03
Pre-escalation (s)	106 (65–148)^a^	52 (30–74)^b^	85 (49–121)^ac^	0.004
Fight duration* (s)	25 (15–35)	51 (16–86)	41 (14–67)	0.35
Winner lesions (n)	30 (11–50)	11(0–24)	12(0–25)	0.29
Loser lesions (n)	66(36–97)^a^	31 (9–53)^b^	36 (12–59)^ab^	0.23

Means with SE for the selected proxy measures of contests costs by treatment group. Values are back-transformed LSmeans with the lower and upper confidence intervals. *Only for contests including a fight; ^a,b^Values lacking a common superscript letter differ by *P* < 0.10.

### Switching strategies between and within contests

Comparing between the three treatments (contest 1; contest 2 control; contest 2 experienced), there was a significant difference between the slopes of the relationship of winner RHP and the duration of the pre-escalation phase. In contest 1, and contest 2 for the experienced dyads, there is no relationship between winner RHP and the duration of the pre-escalation phase, whereas for the contest 2 control group there was a significant negative relationship (Table 4). Table 4 also reveals that for contest 2 control dyads the assessment strategy differs between the pre-escalation phase and the escalation phase, indicative of switching between strategies within a contest. The relationship between loser RHP and injuries on the winner’s body also significantly differed between contests, with a positive relationship in contest 1 and contest 2 dyads with experience, contrasted to the absence of such a relationship in contest 2 control dyads (Table 4), supporting pure self-assessment in the former two types of contest.

**Table 4:** Winner and loser RHP (body weight) in relation to various contest costs for contest 1, contest 2 control (no fighting experience except contest 1), and contest 2 of dyads that received profound fighting experience.

		Contest 1	Contest 2 Control	Contest 2 Experience	*F*_*3,265*_	*P*-value
Contest duration (s/kg)	Winner	10.0	−9.9	10.1	0.38	0.77
	Loser	10.2*	10.2	10.	1.56	0.20
Pre-escalation (s/kg)	Winner	10.2	−9.7*	10.2	2.97	**0.03**
	Loser	10.0	10.4*	−9.9	2.05	0.11
Fight duration^a^ (s/kg)	Winner	−9.6	10.3	−9.6	1.26	0.29
	Loser	10.3	−9.7	10.4	1.04	0.38
Winner lesions (n/kg)	Winner	−8.5	7.9	−6.5	0.70	0.56
	Loser	21.6**	−1.2	14.2*	3.63	**0.01**
Loser lesions (n/kg)	Winner	−10.2	1.5	−16.6	0.94	0.42
	Loser	19.4	1.5	21.8*	2.06	0.11

Values are back-transformed beta estimates for the change in costs per kg of increase in body weight. The *P*-value indicates the significance of the change in the slope between the treatment groups. ^a^Fight duration includes the contests with a fight only (n = 144) opposed to all contests (n = 270). *RHP significantly affects the contest costs by *P* < 0.05; **by *P* < 0.01.

### Influence of sex and aggressiveness on contest costs

Aggressiveness as a personality trait, as determined pre-contest in a resident-intruder test through attack latency, was included in the models as a covariate. Losers that were scored pre-contest as being more aggressive showed a shorter pre-escalation phase in contest 1, meaning a shorter time until the first attack was made (*b* = −1 s pre-escalation/s attack latency (0–600 s), *F*_1,138_ = 16.88, *P* < 0.001).

The sex of the opponents had profound effects on the contest costs in terms of the durations of behaviours and the number of skin lesions, irrespective of weight matching. Contests between two male opponents were most costly, regardless of the age, body weight or experience of the pigs. For example, the average number of skin lesions in male-male contests in contest 2 was 3.7 times greater than in male-female contests and 2.2 times greater than in female-female contests. The details of the sex differences will be published separately to do justice to the many aspects of sex differences in pig contest behaviour.

## Discussion

The aim of this study was to investigate whether pigs, being a highly intelligent mammal, use mutual assessment during a dyadic contest and whether significant experience of fighting alters the assessment strategy. In addition, we investigated whether pigs adopt different assessment strategies in the pre-escalation phase compared to the escalated phase of a contest. From different proxy measures of contests costs, fight duration and skin lesions as a reflection of the number of bites received best reflected the costs accumulated during a contest.

Experience profoundly affected the response to the contest situation, albeit not as expected. Most profoundly, the number of contests that escalated into a fight was reduced by a third in the second contest, irrespective of the level of experience. RHP asymmetric dyads in contest 2 escalated least, which would be in line with mutual assessment, as the inferior individual may decide to retreat without getting into an injurious fight. Applying the appropriate game theory models suggests that mutual assessment was, however, only present in the pre-escalation phase in the control dyads of contest 2. In these contests opponents apparently switched from mutual assessment in the pre-escalation stage to no clear assessment strategy in the escalated phase. Overall, profound experience did not differ from mild experience (control group which had experience of contest 1) in terms of fight escalation but mild experience was more beneficial for the subsequent use of mutual assessment than profound experience.

### The effect of experience

Experience reduced the likelihood of an escalated fight. Both in the experienced and control treatments more encounters were resolved without escalating to fighting in the second contest. Although it seems likely that this was an effect of the experience gained from the first contest, it is not possible to disentangle this from a potential temporal confound because the two contests were staged at different ages, albeit only three weeks apart. The question arises as to how much and what type of experience is necessary to optimise assessment ability.

Experience of fighting, evidenced through skin lesions compared to an unmixed control group, clearly altered aggressive behaviour in the subsequent dyadic contest. Compared to controls, the experienced group showed longer contest durations. However, this was driven by a longer, low cost non-damaging pre-escalation phase, rather than an increase in actual costs as seen from the number of skin lesions. The increased time in investigation and display in the experienced group, together with fewer costs relative to contest duration, is consistent with an enhanced assessment ability. However, testing the formal predictions through the relationship between RHP and contest costs revealed mutual assessment only in the control dyads of contest 2, which had experience of a single contest (contest 1). Consistent with the predictions for mutual assessment, the duration of the pre-escalation stage significantly increased with loser RHP whereas it significantly decreased with winner RHP. The slopes of naïve (contest 1), control and experienced dyads indeed significantly differed from each other. This relationship was, however, only for the pre-escalation phase and only for the control dyads in the second contest. By contrast, for contest 1 and the experienced dyads in contest 2, the results were consistent with predictions for pure self-assessment, with positive relationships between loser RHP and contest costs. Speculating on the differences between the two types of second contests, the results suggest that the intense mixing experience with multiple unfamiliar individuals may have favoured the use of a self-assessment strategy, perhaps due to the costs associated with trying to gain information from a range of aggressively competing conspecifics^3^. This is in contrast to the control dyads that remained housed with familiar conspecifics, a situation that may have favoured the development of enhanced information-gathering skills during low escalation phases and was thus revealed by evidence supporting mutual assessment in the pre-escalation phase.

### Switching strategies

As detailed above, control dyads in contest 2 showed mutual assessment during the pre-escalation phase but not during the phase of escalated mutual fighting where they showed no evidence of any form of assessment. This indicates a switch between strategies in line with previous findings. Hsu *et al*.^27^ showed that killifish (*Kryptolebias marmoratus*) apply mutual assessment during the display phase whereas they switch to self-assessment during escalation. This is in line with the behavioural observations in pigs, where during the pre-escalation phase opponents show mutual investigation^26^ and behaviour such as parallel walking which is said to inform the individual about the opponent’s size^31^, whereas during the escalated phase the aim is to deliver attacks at maximum intensity. The dyads of contest 1 and of contest 2 with fighting experience did spent more time in the pre-escalation phase (as shown in Table 3) but did not show this switching between strategies. The pre-escalation phase can consist of social interactions that, as described above, can assist in mutual assessment, but can also consist of behaviour unrelated to the opponent (e.g. exploring the environment). That mutual assessment was apparent for contest 2 control dyads but not for others may have been due to a different behavioural repertoire in the pre-escalation phase.

### Contest costs

The cost of a contest is an essential measure in the application of game theory models for animal contests. Traditionally the total contest duration is used as a measure of costs. In previous work it was shown that total contest duration can be a poor measure of costs when opponents engage in non-agonistic behaviours during the course of the contest^26^. Indeed, in the current work total contest duration poorly related to the costs that more directly reflect energetic effort and risk of injury. Moreover, the longer contest duration due to a longer non-damaging display phase (pre-escalation phase) does not equate to more actual costs, as shown in contest 2. Fight duration has been suggested as a better measure of costs but this can only be recorded in contests in which a fight occurs. Across species and studies, varying levels of non-escalation have been reported, with the current work having up to 74% of the dyads not escalating into a fight depending upon the treatment group. Non-escalated contests reveal important information on the decision making process of the contestants and therefore suitable measures are required to assess the costs in these contests as well. Moreover, contest duration and fight duration apply to both contestants, implying that the costs would be the same for both winner and loser. In reality the costs for the loser and winner are likely to differ, and measures on the individual are therefore more accurate.

Physiological costs indicated by changes in blood lactate and glucose have previously been used to reflect contest costs for the individual contestants and can be measured regardless of the level of escalation (a.o. refs^32,33^). However, baseline values for lactate and glucose are subject to individual variation and depend upon factors such as time of day and the time of the last meal. In pigs, skin lesions are a direct cost from aggression as they reflect the number of bites received in the contest. Even if no mutual fight occurs, some lesions will appear due to unilateral bites. Skin lesions can therefore be recorded regardless of the occurrence of an escalated fight. We assessed durations of behaviour, glucose and lactate as well as skin lesions as proxy measures of contest costs. From these, fight duration and skin lesions showed the strongest correlations with the other proxy measures and therefore best reflected the contest costs.

### Implications for further research and animal welfare

Animal contests have long been analysed using the traditional approach of correlating winner and loser RHP against contest costs. The analysis of animal contests does however continue to develop profoundly, with advances in the interpretation of models^17^, the required framework to distinguish between models^3^, various manners to statistically analyse animal contests^34^, and the exploration of new factors contributing to RHP (e.g. personality, refs^35,36^). We propose that, as also advocated in ref.^37^, new measures of contest costs that better reflect the metabolic effort and fitness consequences should be considered where relevant. Where species specific measures exist, such as for example skin lesions in pigs and acrorhagial peels in sea anemones (*Actinia equine*; ref.^38^), these may be preferred over traditional proxy measures of contest costs. In addition, the use of total contest duration as a measure of contest costs should be reconsidered, especially for species that spend time in non-agonistic behaviour during a contest.

Aggression is an important animal welfare problem in pig husbandry and research contributing to the understanding of pigs’ assessment abilities during agonistic encounters can inform future efforts to find effective methods of controlling it. The influence of experience, even when brief, reduced the likelihood of an encounter escalating into a fight. Despite the initial costs of fighting, the gained experience may reduce costs on the long term when animals are older and costs are likely to be more severe. Early mixing of unfamiliar pigs to enhance their social abilities has been suggested as a method to reduce aggression as a welfare problem in practice^39^. This has been tested in young piglets from an applied perspective, mainly in terms of farm management strategies, but had never been tested in a game theoretical approach. We are currently investigating the effect of early life experience (at 14 days of age) using the same formal setting which allows animal contest models to be applied. The results are similar to the current study, but with the pigs being nine weeks younger when they receive their experience, the costs to gaining this experience are substantially less^40^. This shows that there can be substantial benefits in allowing animals to gain experience early in life to improve animal welfare.
